# Metabolomics Profiling and Advanced Methodologies for Wheat Stress Research

**DOI:** 10.3390/metabo15020123

**Published:** 2025-02-13

**Authors:** Zhen Liu, Jiahui You, Peiying Zhao, Xianlin Wang, Shufang Sun, Xizhen Wang, Shubo Gu, Qian Xu

**Affiliations:** 1 National Key Laboratory of Wheat Improvement, College of Agronomy, Shandong Agricultural University, Taian 271018, China; huzheng_007@163.com (Z.L.); cornucopiaz@163.com (P.Z.); sun17653475426@163.com (S.S.); 2 Shandong Guocangjian Biotechnology Co., Ltd., Taian 271018, China; yy961328021@163.com (J.Y.); xianlin2015@163.com (X.W.); sdguocangjian@126.com (X.W.)

**Keywords:** abiotic stress, biotic stress, metabolomics, omics, wheat

## Abstract

Metabolomics is an omics technology that studies the types, quantities, and changes of endogenous metabolic substances in organisms affected by abiotic and biotic factors. Background/Objectives: Based on metabolomics, small molecule metabolites in biological organisms can be qualitatively and quantitatively analysed. This method analysis directly correlates with biological phenotypes, facilitating the interpretation of life conditions. Wheat (*Triticum aestivum* L.) is one of the major food crops in the world, and its quality and yield play important roles in safeguarding food security. Methods: This review elaborated on the significance of metabolomics research techniques and methods in enhancing wheat resilience against biotic and abiotic stresses. Results: Metabolomics plays an important role in identifying the metabolites in wheat that respond to diverse stresses. The integrated examination of metabolomics with other omics disciplines provides new insights and approaches for exploring resistance genes, understanding the genetic basis of wheat metabolism, and revealing the mechanisms involved in stress responses. Conclusions: Emerging metabolomics research techniques to propose innovative avenues of research is important to enhance wheat resistance.

## 1. Introduction

**Figure 1:**
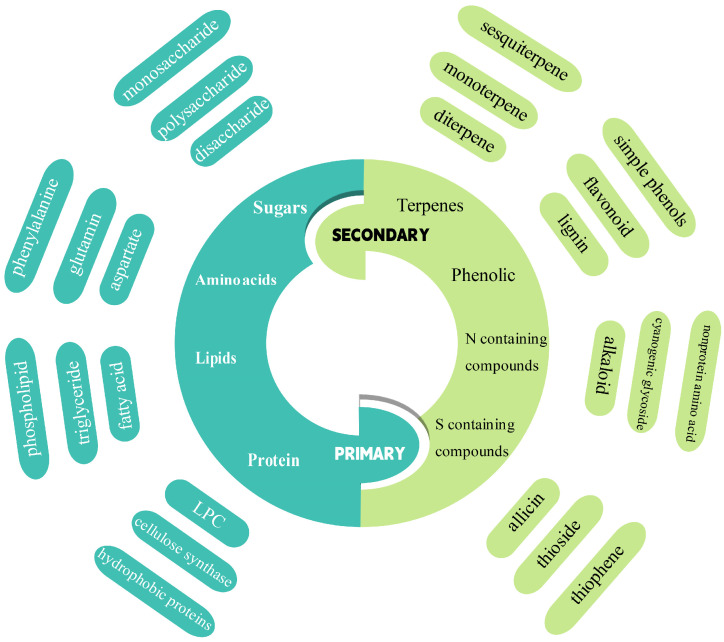
Classification of primary and secondary metabolism in wheat. Each class is subdivided into several subclasses that carry out specific plant functions depending on the conditions. Most of the responses of wheat under stress will be manifested in metabolite changes, mainly in pathways containing phenolics, terpenes, lipids, and amino acids.

Metabolomics has emerged as an important omics technology following proteomics, transcriptomics, and genomics and is an important branch of systems biology. Metabolomics was first proposed in 1998 [1,2]. Genomics studies the gene structure, function, evolution, and localization of all organisms. Proteomics comprehensively analyses protein expression patterns, modification status, structural functions, and interactions. Metabolomics is the study of the composition and changes of metabolites in organisms. Compared to other omics fields, metabolomics can reveal changes in metabolites in organisms affected by genetic or environmental factors and establish a direct correlation with the phenotype of the selected organism [3]. Metabolomics has recently received much attention for its useful applications in different fields, such as medicine, animal husbandry, food science, and botany [4,5]. Metabolites are the final products of gene expression. It is estimated that the total number of metabolites in plants exceeds 200,000. Primary metabolites are essential for growth and development and maintaining life activities, whereas secondary metabolites are byproducts of primary metabolites that participate in the response to the environment and are associated with stress (Figure 1).

Wheat is one of the world’s most important food crops, and its production is related to global food security. Cultivated wheat includes diploids, tetraploids, and hexaploids (x = 7). The most cultivated crop is hexaploid wheat (*Triticum aestivum* L.) (AABBDD), which can be processed into steamed buns, noodles, biscuits, bread, and other foods and occupies an important position in the human diet [6]. Great achievements have been made in wheat genome research, and detailed mapping of reference genome sequences of diploid, tetraploid, and hexaploid wheat has been completed [5,7,8]. Genome-wide association analysis of wheat has promoted research on genetic evolution, genome structure, and variation. Metabolomic analysis is utilized to reveal the changes in metabolites within cells or organs that are impacted by genetic or environmental factors, and these changes are more readily linked to phenotypic analyses than to genomics, transcriptomics, and proteomics [9,10,11,12]. Recently, metabolomics has been employed to study the mechanisms of plant responses to biotic and abiotic stresses [10,13]. At the same time, metabolomics combined with other methods, such as quantitative genetic transcriptomics and gene editing, has revolutionized plant genetic improvement [14].

Metabolomics progress depends on metabolite detection, high-throughput data analysis technologies, and shared database availability. The types of databases mainly include “general chemical” databases, such as ChemSpider (http://www.chemspider.com/, accessed on 8 February 2025) [15] and PubChem (https://pubchem.ncbi.nlm.nih.gov/, accessed on 8 February 2025) [16,17]; “Pathways” databases, such as Biocyc (http://www.biocyc.org/, accessed on 8 February 2025) [18], KEGG https://www.kegg.jp/, accessed on 8 February 2025) [19,20], and Reactome (http://www.reactome.org/, accessed on 8 February 2025) [21]; “Metabolites” databases, such as HMDB (https://hmdb.ca/, accessed on 8 February 2025) [22], LMSD (https://www.lipidmaps.org/resources/databases/index.php, accessed on 8 February 2025) [23], Pmhub (https://pmhub.org.cn/, accessed on 8 February 2025) [24], and GMD (http://gmd.mpimp-golm.mpg.de/, accessed on 8 February 2025) [25]; and “Mass Spectrometric” databases, such as NIST (https://www.nist.gov/, accessed on 8 February 2025) [26], METLIN (http://metlin.scripps.edu/, accessed on 8 February 2025) [27,28], and Massbank (http://www.massbank.jp/, accessed on 8 February 2025) [29]. According to statistics, approximately 5000 plant metabolites are in public databases, far from the 200,000 kinds of natural plant metabolites in the biological world.

Metabolites play a vital role in wheat’s stress physiology and nutritional quality. Metabolomics is the key omics method to study the types, distribution, and biological functions of metabolites. More than 2000 metabolites have been identified in wheat, including hormones, flavonoids, and polyphenols that may control or influence the resistance physiology [30]. However, the metabolic pathways and regulatory networks are not as well understood as in other plants due to the complexity of the wheat genome. With the advent metabolite genome-wide association study (mGWAS) and metabolite quantitative trait locus (mQTL) analyses, combining metabolomics with genomics, transcriptomics, and proteomics would help identify candidate genes and elucidate metabolic pathways in stress physiology.

**Figure 2:**
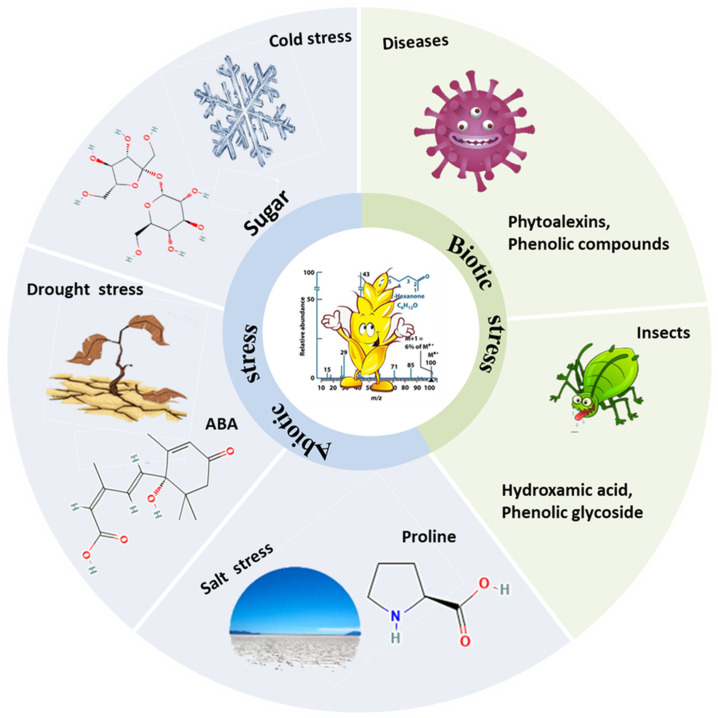
Wheat responds to different environmental stresses through different mechanisms. Wheat produces different metabolites in response to certain stressors, whereas some metabolites are routinely generated in stressful situations, others show a high level of specificity.

This review focuses on applying metabolomics to improve wheat stress tolerance to abiotic and biotic stresses (Figure 2, Table 1). Additionally, we provide a brief overview of the innovative approaches, strategies, and data processing procedures related to agricultural productivity in metabolomics.

## 2. Metabolomic Applications for the Study of Wheat Stress Tolerance

Metabolomics enhances wheat stress resistance in two ways: (1) Integrated metabolomics and other omics analyses (transcriptomics, genomics, etc.) are utilized to identify resistance genes that regulate stress tolerance in wheat. The resistance of wheat to different factors can be genetically modified using molecular techniques. (2) Based on metabolomics, metabolites in wheat that respond to stress and enhance wheat stress tolerance can be screened through the exogenous application of selective chemicals (Figure 2). Many important metabolites in wheat respond to environmental stress conditions. For example, proline is critical for osmotic adjustment and maintains cellular function and structure under drought and salt stress conditions [53,54,55]. Polyamines regulate various physiological processes, including ROS scavenging and membrane stability [56,57]. Amino acids such as arginine and lysine contribute to the synthesis of stress-related proteins [58,59,60]. Phenolic compounds act as antioxidants and counteract oxidative stress [61]. Lipids and fatty acids impact membrane fluidity and integrity under stress conditions [62]. Many soluble sugars, such as glucose and sucrose, act as osmoprotectants, preventing cellular dehydration under stress [63,64]. Various secondary metabolites, such as alkaloids and flavonoids, improve plant defensive strategies and resilience against stress [65,66]. Nitrogen-containing compounds such as nitric oxide and gamma-aminobutyric acid facilitate stress signalling and adapt to stress [67]. In short, metabolite regulation is fundamental for coordinating complex plant adaptations to various stressors.

### 2.1. Abiotic Stresses

The primary factors that impact plant growth and ultimately crop yield are abiotic stresses, which include drought, salinity, extreme temperature, waterlogging, heavy metals, and low-temperature damage [11,68,69]. Endogenous metabolic homeostasis is disrupted and a new metabolic balance established during stress adaptation [13]. Metabolomic technology can identify metabolites with significant differences under stress conditions, thereby revealing key metabolites and pathways that may play roles in adaptive resistance. Numerous research and review papers have contributed to elucidating the roles of metabolites in plant growth, development, and environmental stress conditions. This review focuses on metabolites in wheat, citing recent articles for comprehensive insights.

#### 2.1.1. Low-Temperature Stress

Low temperature adversely affects wheat yield. Wheat breeders need to produce or improve the resistance of wheat cultivars to freezing. Changes in membrane lipids have been recognized as crucial elements in enhancing freezing tolerance. Following cold acclimation of the three wheat varieties, metabolic data indicated that the plasma membrane changed the lipid content significantly, and the ratio of free sterols to glycolipids increased in direct proportion to the degree of freezing resistance [32]. Cheong et al. employed a combination of targeted metabolomics and lipidomics methods, along with gas chromatography–triple quadrupole mass spectrometry (GC-QQQ-MS) technology, to assess the metabolite and lipid levels in the flag leaves of both cold-sensitive and cold-tolerant wheat varieties. The results revealed that the accumulation of osmotic substances, such as sugar, glucose, putrescine, and shikimic acid, indicated low-temperature stress tolerance [31]. Lv et al. analysed the transcriptome and metabolome of cold-sensitive mutant wheat and detected 88 differential cumulative metabolites, including p-coumarinyl putrescine, an alkaloid; D-proline, an amino acid; betaine; and chlorogenic acid, a derivative of phenolic acid. Comprehensive analysis revealed that the cold resistance of wheat is closely related to 13 metabolites and 14 key enzymes in the flavonol biosynthesis pathway [33]. Recently, two wheat varieties were investigated using targeted phytohormone metabolomics, revealing a correlation between auxin, jasmonic acid, cytokinin, and cold resistance. These findings lay the foundation for a deeper understanding of the molecular mechanisms through which endogenous plant hormones regulate frost resistance in plants [34]. A genome-wide association study identified two critical genes for low-temperature resistance in wheat. This study also elucidated the molecular mechanism of chilling tolerance via transcriptomics, indicating that it may increase JA (jasmonic acid) content by promoting the accumulation of α-linolenic acid and repressing the degradation of JA. Targeted metabolomics confirmed that the content of JA in transgenic lines significantly increased, and exogenous spraying of JA significantly improved the low-temperature resistance of wheat [35].

#### 2.1.2. Drought Stresses

Drought is the primary environmental factor affecting wheat productivity. Targeted metabolomics with GC-MS was conducted on the flag leaves of different wheat varieties under drought stress. The results revealed a significant increase in proline and tryptophan contents under drought stress. The drought-tolerant varieties exhibited a decrease in organic acid content, while succinic acid, aspartic acid, and trehalose levels significantly increased, indicating their crucial role in the energy production pathway [37]. *Triticum dicoccoides* Korn was found to be a genotype with high drought tolerance and has great potential as a genetic model system for verifying metabolomics–genomic networks. The metabolic response of wheat to drought stress induced by arbuscular mycorrhizal fungi was analysed using UHPLC-ESI/QTOF-MS (ultrahigh-performance liquid chromatography–electrospray ionization/quadrupole time-of-flight mass spectrometry). The results revealed significant stimulation of the brassinosteroid biosynthesis pathway in the roots of inoculated wheat plants. The results showed that the stimulation of the brassinosteroid biosynthesis pathway was particularly obvious in inoculated wheat roots, which supported the hypothesis that the brassinosteroid biosynthesis pathway was involved in enhancing the plant response to water stress and regulating oxidative stress conditions [38]. The screening of drought-tolerant genotypes plays a major role in the breeding of drought-resistant wheat varieties. Non-targeted metabolomics was conducted via liquid chromatography-mass spectrometry (LC-MS) to detect the metabolite levels of drought-tolerant and drought-sensitive wheat varieties. Lysine, arginine, methionine, proline, and tryptophan accumulation in drought-tolerant varieties could be used as potential biomarkers for screening drought-tolerant genotypes [39,40,41]. Furthermore, a recent study on wheat demonstrated that α-tocotrienol, linoleic acid, and L-leucine could induce comprehensive and systematic drought resistance during seed germination. This effect is achieved by activating the mTOR-ABA signalling pathway and promoting the interaction of various hormones [42].

#### 2.1.3. Saline–Alkali Stresses

Salinization is characterized by high pH levels, an environmental threat to agricultural systems. Salt and alkali stress exerts detrimental effects on wheat metabolism and hinder the accumulation of essential nutrients. GC-MS technology was used to detect changes in metabolites in the leaves and roots of wheat plants under salt and alkali stress. The findings revealed distinct metabolic changes caused by salt and alkali stress. Under alkali stress, the increase in Ca^2+^ levels in roots activates the salt overly sensitive (SOS) pathway and Na^+^ excretion mechanism, mitigating Na+ damage [70]. On the other hand, salt stress prompts a metabolic shift towards gluconeogenesis, resulting in increased sugar content to alleviate osmotic stress. These findings shed light on the physiological responses and adaptive strategies of wheat plants to counteract salt and alkali stress [41]. Salt stress reduces plant growth and productivity due to high levels of sodium ion accumulation. The inoculation of plant-growth-promoting rhizobacteria (PGPR) into plant roots reduced salt stress [71]. Aside from the increase in plant biomass under stress conditions, phenylalanine metabolism, caffeine metabolism, galactose metabolism, and terpenoid–quinone biosynthesis were significantly altered, which may play an important role in salt stress conditions in wheat seedlings. Furthermore, the correlation analysis excluded the possibility that after PGPR inoculation, changes in amino acid, porphyrin, and chlorophyll metabolism primarily improved wheat growth under salt stress conditions [44].

### 2.2. Biotic Stresses

Biotic stresses refer to the stress created by biological organisms, including pathogens, pests, weeds, and rats. In particular, pathogens and pests significantly reduce annual agricultural yields. The impact of biotic stress on crop production or quality is contingent upon the diverse agroclimates seen in various regions. Integrating modern genomics, phenomics, and metabolomics approaches with next-generation sequencing has made it easier to accurately locate and select resistance genes for biotic stress resistance in wheat breeding.

#### 2.2.1. Wheat Diseases

Pathogens including fungi, oomyces, bacteria, and viruses can directly or indirectly impact wheat growth, leading to restricted growth, decreased yield, or even death [72]. Fusarium head blight (FHB) is a destructive fungal disease caused by the fungus *Fusarium graminearum*. To observe the differences in metabolites, NMR (nuclear magnetic resonance)- and GC-MS-based metabolic profiling were conducted on four strains of *Fusarium*. The results revealed that the nutritional environment has a greater impact on fungal metabolism than does the genotype [45]. Using untargeted metabolic profilometry and the LC-ESI-LTQ-Orbitrap technique, FHB in wheat decreases grain production and contaminates grains with trichothecene mycotoxins. The inheritance of host resistance to FHB is quantitative, and more than 100 quantitative trait loci (QTLs) have been successfully identified. Untargeted metabolomic profiling was used to investigate the resistance mechanisms of tolerant and susceptible plants. This resistance was caused by the accumulation of resistance-related metabolites from the phenylpropanoid pathway. These metabolites hinder the development and progression of infection by increasing the thickness of the host cell wall and reducing pathogen growth and subsequent trichothecene production. In a recent study, researchers employed LC-MS-targeted techniques to identify the production of flavonoids in wheat induced by pathogens. The structure of this compound was subsequently determined using 1D and 2D NMR analysis, confirming that it is an isoflavonoid [47]. This substance mediates important interactions with plant-associated microorganisms [73]. Farahbakhsh detected the metabolic characteristics of wheat-resistant or wheat-susceptible cultivars to stripe mosaic virus (WSMV) via UPLC-QTOF/MS technology [49]. These characteristics were then evaluated to determine the resistance of wheat to WSMV at different temperatures. Wheat powdery mildew (BGT) is a fungal disease that poses a serious threat to the global development of wheat agriculture. Recent metabolomics studies using LC-MS were applied to two varieties with different resistances. It was confirmed that the contents of levodopa and tyramine were correlated with wheat resistance to powdery mildew and can be used as metabolic markers for genetic improvement [48]. Furthermore, this study provides a new foundation for breeding wheat varieties resistant to powdery mildew. In another study, following powdery mildew inoculation, metabolome analysis indicated the flavone and flavonol biosynthesis pathways as the most significantly enriched [74]. The metabolic profile suggested that the phenylalanine pathway might link flavonoid production to leaf rust resistance in the salicylic acid-treated leaves, while alanine, aspartate, and glutamate metabolism might control the production of other amino acids to the enhance pathogen stress response in the jasmonic acid-treated leaves [75]. Metabolites responsive to Fusarium crown rot (FCR) infection showed 102 metabolites with significant changes, mainly in the flavonoid, phenolic acid, amino acid, and derivative classes. Proline betaine, lauric acid, ribitol, and arabitol were stably induced by Fusarium pseudograminearum (Fp) infection and may have important roles in FCR resistance [76].

#### 2.2.2. Wheat Insect Attack

Plants use secondary metabolites as defensive mechanisms to evade or adjust to the dynamic environment. Secondary metabolites, including alkaloids, glycosides, phenolic compounds, and terpenoids, are retained in specialized structures such as trichomes, resin ducts, and latex [77]. Modern technology can reveal the structural and functional characteristics and biosynthesis pathways of these metabolites. An enhanced understanding of molecular and enzymatic regulation will facilitate the investigation of secondary metabolites in modern approaches for managing pests. The aphid salivary proteins trigger defence responses in wheat and, through salivary gland RNA-seq, four chemosensory protein genes were identified. This study utilized LC-MS/MS technology to detect salicylic acid in wheat leaves to elucidate the mechanism by which the chemosensory protein SmCSP4 functions. SmCSP4 is secreted into wheat leaves during aphid feeding, activating salicylic acid (SA)-mediated plant defence responses and reducing aphid performance by inhibiting feeding behaviour [50]. Non-targeted and targeted metabolomic analysis using LC/Q Exactive/MS technology was used to study the metabolites on aphid-affected leaves, and the results showed that benzoxazine synthesis was partially correlated with aphid reproduction [51]. A combined strategy of transcriptomic and metabolomic analysis has been carried out in wheat seeds following blossom midge (*Sitodiplosis mosellana Géhin*) feeding, which revealed that differentially expressed genes and accumulated metabolites were primarily enriched in several primary and secondary metabolic pathways, including phenylpropanoid biosynthesis, flavonoid biosynthesis, and phenylalanine biosynthesis [52].

## 3. Advancements in Metabolomics Research

**Figure 3:**
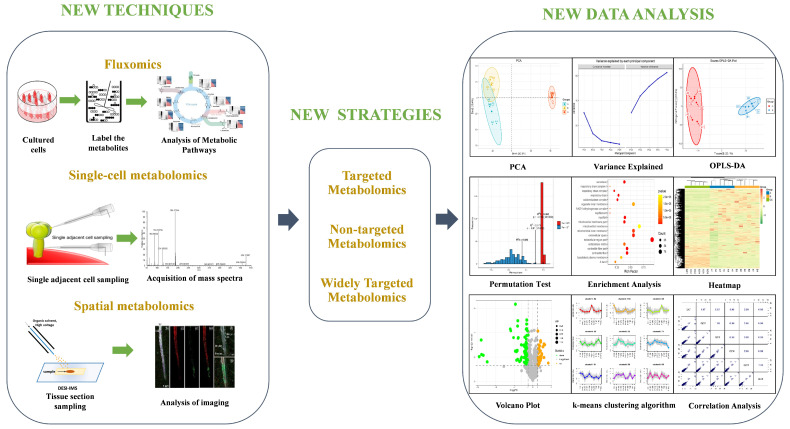
Metabolomics advancements comprise a range of approaches, tactics, and data processing procedures. The introduction of advanced techniques such as metabolic flux analysis, single-cell metabolomics, spatial metabolomics, and other developing technologies has expanded metabolomics data. Furthermore, the use of novel metabolomics approaches, particularly targeted metabolomics, has improved the accuracy and practicality of metabolite identification. Subsequently, these metabolomics datasets are examined by employing innovative data processing techniques.

Metabolomics has undergone significant advancements in recent years, particularly in fluxomics, spatial metabolomics, and single-cell metabolomics. These techniques have provided researchers with valuable tools for studying metabolic pathways, understanding the spatial distribution of metabolites, and exploring cellular heterogeneity at the single-cell level (Figure 3).

### 3.1. Spatial Metabolomics

Spatial metabolomics is an interdisciplinary field that integrates metabolomics with mass spectrometry imaging technology. It enables the transformation of omics information from a two-dimensional to a three-dimensional level and the precise characterization of the species, content, and spatial distribution of metabolites within organisms. In spatial metabolomics, commonly employed mass spectrometry imaging techniques include DESI (desorption electrospray ionization) and MALDI (matrix-assisted laser desorption ionization). These techniques enable rapid analysis of a sample’s composition, spatial distribution, and relative abundance of metabolites. For example, Perdian et al. used spatial metabolomics to identify and accurately determine the locations of structural isomers of different flavonol glycosides in Arabidopsis petals [78]. In a recent report, to uncover the multigenerational effects of an elevated atmospheric CO_2_ concentration (eCO_2_) on wheat, AFADESI-MS (ambient ionization-based desorption electrospray ionization mass spectrometry) spatial metabolomics was carried out to investigate the spatial distribution of amino acids and organic acids in wheat grains [79]. A MALDI-MS system was applied for subcellular-level mass spectrometry imaging of maize leaves, which revealed the asymmetric metabolic characteristics of various cell types [80]. The symbiotic relationships between leguminous plants and soil bacterial rhizobia are distinct and mutually beneficial. The MALDI method was used to comprehensively observe the differences in the composition and distribution of metabolites during the alfalfa nitrogen fixation process [81]. A combined strategy using UPLC-QTOF-MS- and MALDI-TOF-MSI-based metabolic analysis has been carried out in *Dendrobium nobile Lindl* to investigate the spatial distributions and biosynthetic pathways of alkaloids and sesquiterpenes [82]. Using spatial metabolomics has shown promising potential for development in many plant species, such as wheat, maize, and soybean.

### 3.2. Fluxomics

Fluxomics is a modern topic of omics research that quantifies the rates of all internal fluxes in the core metabolism of living systems. Fluxomics involves the collection of data from many omics disciplines, providing a comprehensive view of molecular interactions. Fluxomics has emerged as a very significant method for studying metabolic characteristics. The use of ^13^C-labelled molecules for metabolic flux analysis is becoming more prevalent for observing metabolic pathways and investigating the associated network of gene–RNA and protein–metabolite interactions [83]. Isotope-labelled (^13^C and ^15^N) precursor substances were used to cultivate the plants. Then, metabolic changes were dynamically monitored through continuous sampling during the synthesis and degradation of the organism’s substances. This technology enables the identification and quantification of metabolic pathways and the flow of metabolites, overcoming limitations in quantifying metabolites over a specific time period in cells or tissues. The leaves of *Arabidopsis thaliana* were labelled with ^13^CO_2_, and dynamic metabolic changes in photosynthetic end products and intermediate products were detected in response to bright light using GC-MS and LC-MS techniques. These analytical methods successfully constructed metabolic flow analysis models, encompassing key metabolic pathways such as the Calvin cycle, photorespiration, and amino acid biosynthesis [84]. Metabolic flux analysis has become a powerful tool for studying intricate changes in photosynthetic flux in terrestrial plants [85]. Furthermore, it enables a comprehensive understanding of the dynamic alterations occurring in the carbon metabolism network of plants [86]. Overall, metabolic flux analysis provides valuable insights and offers a promising approach for advancing crop improvement efforts.

### 3.3. Single-Cell Metabolomics

Single-cell metabolomics allows for the simultaneous detection of a wide range of metabolites from individual cells without preselection or labelling. This technique permits the mapping of phenotypes at the single-cell level. This is a new field compared to other single-cell methods, such as single-cell sequencing and proteomics. The capabilities of single-cell metabolomics have been recognized via many biological applications in several fields. The field of single-cell metabolomics is advancing quickly, with a growing number of research groups, improved methods for cell collection and ionization, sophisticated tools for data processing, and diverse applications to address significant biological and environmental questions. Furthermore, it demonstrates exceptional ingenuity in addressing obstacles related to limited sample sizes, the diverse array of metabolite types, and extensive datasets [87]. Single-cell metabolomics utilizes various ion sources, including electrospray ionization (ESI), laser desorption ionization (LDI), and secondary ionization mass spectrometry (SIMS), to ionize small molecules. These ions are then introduced into the mass spectrometer for imaging and detection. In component analysis, each cell generates only one or a few mass spectrograms to display metabolite components. On the other hand, imaging analysis involves the collection of multiple mass spectrograms from different regions of a single cell, which are then processed into images that provide spatial information about the distribution of metabolites [88]. The single-cell metabolomics method was used to absorb the cytoplasm of individual cells from different tissues of geranium in situ, and nano-ESI technology was used for detection [89]. LAESI (laser ablation electrospray ionization mass spectrometry) technology was utilized to determine the microscopic anatomy and analyse onion epidermal cells and investigate the metabolites involved in compartmentalization in the subcellular domain [90]. Single-cell metabolomics has yet to be utilized in wheat metabolomics research and has great potential for exploration. These advanced technologies show vast prospects for application in agricultural production.

## 4. Combined Analysis of Metabolomics and Other Omics (Genetics, Transcriptomics, Proteomics)

Multiomics, combining metabolomics with genomics, transcriptomics, or proteomics, will make a network clearer, from responsive genes to transcribed mRNA and then from translated proteins to final metabolites [91,92]. In this way, key functional gene expression patterns and pathways are explained more accurately and comprehensively. Combinations of genetic approaches such as quantitative trait loci (QTL) and genome-wide association study (GWAS) with metabolomic profiling have been widely applied in order to identify the functional genes underlying the content variation of metabolites as powerful tools in the large-scale identification of candidate genes. These methodologies are termed as mQTL (metabolite QTL) or mGWAS (metabolite GWAS), which have been widely utilized in major crops, including tomato [93], maize [94], rice [95], barley [96], and tea [97].

Based on previous research, certain genes within the quantitative trait locus (QTL) region were shown to enhance resistance through the reinforcement of cell wall structure, while others achieved resistance by mitigating the toxicity of deoxynivalenol (DON) [98]. In a study focused on wheat resistance to stripe rust, transcriptomic analysis revealed an increase in the expression of the chitinase-related gene *atchi8* in leaves treated with salicylic acid. Additionally, metabolomics analysis suggested a potential link between the phenylalanine pathway and the production of flavonoids, thereby enhancing leaf rust resistance and bolstering pathogen resistance [75]. The response mechanism of winter wheat Jing 411 to cold stress was analysed. It was found that abscisic acid, JA plant hormone signalling, and the proline synthesis pathways might play important roles in freezing tolerance [44]. Gunnaiah et al. conducted proteomics and metabolomics analysis on wheat near-isogenic lines (NILs) that were infected or not infected with *F. graminearum*. The aim of the study was to identify and determine the response pathway of wheat to pathogens [46]. Building upon a similar concept, Michaletti et al. analysed two spring wheat varieties, Bahar and Kavir, to elucidate the comprehensive biochemical network underlying the response of leaves to water stress [39]. These findings present a comprehensive framework that enhances our understanding of the regulatory mechanisms governing the response of plant cells to stress. Chen et al. performed an extensive targeted metabonomic analysis of 182 wheat samples and detected 805 metabolites from the grains. The relative content of these metabolites across the 182 wheat germplasms was further analysed and a metabonomic association study (mGWAS) conducted using 14,646 polymorphic SNP markers. A total of 1098 mGWAS association sites were detected, focusing on analysing the functions of two candidate genes associated with wheat flavonoids, and a flavonoid-modified metabolic pathway was successfully identified [99].

mGWAS is a prominent method utilized to analyse the correlation between metabolome data, such as phenotypes, and genotype data. This approach enables researchers to investigate the associations between genetic variations and metabolic profiles, thereby shedding light on the underlying genetic mechanisms influencing metabolic traits. Due to the enhanced resolution of metabolome data in identifying phenotypes, mGWAS analysis offers greater accuracy in association analysis than traditional GWAS analysis [100]. Based on mGWAS, chemical-tag-based semi-annotated metabolomics is a faster and more unbiased approach for obtaining candidate genes. Zhu et al. analysed the enrichment features of secondary fragments of 391 wheat leaf metabolites. They further developed a rapid quantification method for 1,483 metabolites in wheat leaves that contain at least one characteristic fragment, termed semi-annotated metabolomics. Finally, the study applied semi-annotated metabolomics to a natural wheat population for association analysis. A total of 499 candidate genes were efficiently screened based on the connections between these modification groups and gene functional annotations [101]. Comprehensive multiomics analysis has emerged as a powerful tool for understanding biological mechanisms, analysing gene functions, and supporting breeding efforts.

Multiomics is a powerful tool to investigate the mechanism of resistance to abiotic and biotic stresses. The resistance responses and transcriptional and metabolic changes were integrated and key components of the defence pathways and candidate genes could be identified. Wheat varieties better adapted to specific environmental conditions would be developed, with the understanding of genetic bases and a regulation network leading to improvement of productivity and sustainability. Moreover, the accumulation of whole-genome sequencing, transcriptomics, metabolomics, and epigenomics data has led to a paradigm improvement in wheat research. Multiomics has speeded up the research on wheat domestication and diversification and was further integrated and upgraded to Pan-Omics [102].

## 5. Conclusions and Future Perspectives

Under the new climate patterns, wheat might face extreme temperatures, drought, salinization, and unpredictable weather events. The drastic climate change and epidemic of new pathogens might have great impact on the interaction patterns between wheat and stress. Incorporating genetic diversity and advanced biotechnologies is promising to create resilient wheat with stable yields under adverse conditions. Improving adaptability of wheat is vital for ensuring food security and protecting farmers’ interests. With respect to the stress resistance mechanisms in wheat, metabolomics has made significant progress and demonstrated its valuable applications.

Traditional metabolomics measures metabolites in tissues or cells at specific time points. However, new detection strategies, such as metabolic flux analysis, single-cell metabolomics, and spatial metabolomics, have emerged, enabling multimode and multidimensional metabolomics analysis of samples. These innovative approaches provide more detailed and reliable information for revealing the mechanisms of organisms. Moreover, integrating metabolomics with other “-omics” disciplines can help us better understand wheat’s metabolic diversity and potential genetic variations in response to stress. However, metabolomics faces many challenges. For instance, metabolite accumulation was significantly influenced by epigenetic modifications. The problems require continuous efforts and advancements in methodology and technology.

In recent years, metabolomics has evolved from quantifying known metabolites to exploring the metabolic pathways involving unknown metabolites and their modified derivatives. The scope of differentially abundant metabolite comparisons has also expanded from different varieties within the same species to different species altogether. This has given rise to new analyses, such as comparative and evolutionary metabolomics. With advancements in detection technology, analysis methods, and the expansion of databases, metabolomics is poised to play an increasingly vital role in wheat stress research and other areas such as quality and functional improvement.

## Figures and Tables

**Table 1:** Application of metabolomics under different environmental stress conditions.

Application	Wheat Organs	Metabolites and Pathway	Detection Strategy	Detecting Technique	References
Abiotic Stress					
Low-temperature stress	Flag leaves	Glucose, putrescine, and shikimate	Targeted	GC-QQQ-LS	[31]
	Seedlings	Sterols	Targeted	GC-MS	[32]
	Leaves	Flavonol biosynthesis pathway, P-coumaroyl putrescine of alkaloids, D-proline betaine of amino acids and derivatives, Chlorogenic acid	Non-targeted	LC-MS	[33]
	Tillering nodes	Auxin, jasmonic acid, cytokinin	Targeted	LC–MS/MS	[34]
	Seedlings	Jasmonic acid	Targeted	HPLC	[35]
Drought stress	Flag leaves	Amino acids, organic acids	Targeted	GC-MS	[36]
	Flag Leaves, root	Sugars, amino acids, organic acids	Targeted	GC-MS	[37]
	Root	Brassinolide biosynthesis pathway	Non-targeted	UHPLC-ESI/QTOF-MS	[38]
	Leaves	Amino acids, organic acids and sugars	Non-targeted	LC-MS	[39]
	Flag leaves	Amino acids, organic acids and sugars	Non-targeted	LC-HRMS	[40]
	Leaves	Polyphenols and amino acids	Non-targeted	UPLC-MS	[41]
	Seedlings	α-Tocotrienol, linoleic acid, and L-leucine, mTOR-ABA signal pathway	Targeted	LC-MS	[42]
Saline–alkali stress	Roots, Leaves	SOS response; gluconeogenic pathway	Non-targeted	GC-TOF/MS	[43]
	Seedlings	Galactose metabolism, phenylalanine metabolism, caffeine metabolism, ubiquinone, glutathione metabolism and other terpenoid quinone biosynthesis	Non-targeted	LC-MS	[44]
**Biotic stress**					
Fusarium head blight	Leaves	Glycerol, mannitol, γ-aminobutyric acid	Non-targeted	1H NMR and GC-MS	[45]
	Spikes	RR phenylpropanoids	Non-targeted	LC-ESI-LTO-Orbitrap	[46]
	Spikes	Isoflavonoid	Non-targeted	1D and 2D NMR	[47]
Powdery mildew	Leaves	Levodopa, tyramine	Non-targeted	LC-MS	[48]
Wheat Streak Mosaic Virus	Leaves	Amino acid, lipids and alkaloids	Non-targeted	UPLC-QTOF/MS	[49]
Aphid	Leaves	Salicylic acid	Targeted	LC-MS/MS	[50]
Aphid	Leaves	Benzoxazinoids	Non-targeted and targeted	LC/Q-Exactive/MS	[51]
Blossom midge	Seeds	Phenylpropanoid biosynthesis, flavonoid biosynthesis, phenylalanine biosynthesis	Non-targeted and targeted	LC-MS	[52]

Non-targeted = Non-targeted metabolomics; Targeted = Targeted metabolomics.

## Data Availability

No new data were created or analyzed in this study. Data sharing is not applicable to this article.
